# Factors Associated With Professional Quality of Life Among Exposed Nursing Staff to the COVID‐19 Patients

**DOI:** 10.1155/nrp/1612798

**Published:** 2026-04-29

**Authors:** Faiza Abou El-Soud, Wejdan Shaqiqi, Sarah Aldehimy, Fadwa Alsawelem, Ethar Alnajdi, Maha Faqihi

**Affiliations:** ^1^ College of Nursing, King Saud Bin Abdul Aziz University for Health Sciences (KSAU-HS), Riyadh, 14611, Saudi Arabia, ksau-hs.edu.sa; ^2^ King Abdullah International Medical Research Center (KAIMRC), Riyadh, 11481, Saudi Arabia, kaimrc.med.sa; ^3^ Ministry of National Guard Health Affairs (MNGHA), Riyadh, 11426, Saudi Arabia

**Keywords:** COVID-19, nurses, pandemics, professional quality of life

## Abstract

**Introduction:**

The global impact of COVID‐19 resulted in a negative influence on the professional quality of life of healthcare workers, particularly nurses.

**Objectives:**

To investigate the level and factors impacting the professional quality of life dimensions of nurses having contact with COVID‐19 patients.

**Methods:**

A cross‐sectional, correlational, descriptive design was conducted using a purposive sample. A total of 200 nurses who had provided direct care to COVID‐19 patients were selected from various wards across two governmental hospitals, Riyadh, Saudi Arabia. Data were gathered via self‐report surveys using the Professional Quality of Life Scale and the Multidimensional Scale of Perceived Social Support and analyzed using multivariate regression models. The STROBE checklist for cross‐sectional studies was adhered.

**Results:**

The overall professional quality of life was moderate (*M* = 90.13, SD ± 9.00). Predictor factors of compassion satisfaction were gender (*β* = 0.160, *p* = 0.021), living arrangements during the COVID‐19 pandemic (*β* = 0.139, *p* = 0.049), family infection due to work (*β* = −0.183, *p* = 0.044), number of COVID‐19 patients cared for per shift (*β* = −0.187, *p* < 0.001), and testing positive for COVID‐19 (*β* = −0.372, *p* < 0.001). For secondary traumatic stress, age (*β* = 0.169, *p* = 0.043), gender (*β* = 0.156, *p* = 0.035), family fear of COVID‐19 transmission (*β* = 0.281, *p* < 0.001), and testing positive for COVID‐19 (*β* = −0.070, *p* = 0.033) were significant factors. The factor contributing to burnout was testing positive for COVID‐19 (*β* = −0.275, *p* < 0.001). Furthermore, perceived social support positively influenced compassion satisfaction but negatively impacted secondary traumatic stress and burnout.

**Conclusion:**

The professional quality of life dimensions of nurses was significantly influenced by various personal‐, social‐, and occupational‐related predictors.

**Implications for the Profession and/or Patient Care:**

Nurses experiencing higher CS are more likely to deliver high‐quality care. Conversely, BO and STS do not support patient safety and care continuity.

**Reporting Method:**

The STROBE checklist for cross‐sectional studies was adhered.

**Patient or Public Contribution:**

Societal need to support frontline healthcare workers, which ultimately benefits public health outcomes and pandemic resilience.

**Summary**
◦ What Does This Paper Contribute to the Wider Global Clinical Community?
• The study finding delivers valuable insights into professional quality of life (ProQOL) among nurses during the Coronavirus Disease 2019 (COVID‐19) pandemic.
• The finding indicate that the secondary traumatic stress (STS) and burnout (BO) were prevalent among nurses caring for COVID‐19 patients.
• It highlights how personal, social, and occupational factors shape compassion satisfaction (CS), BO, and STS.
• Promoting CS is essential for enhancing care quality, while mitigating BO and STS is vital for maintaining patient safety and continuity of care.
• The finding highlights how external support for frontline nurses, especially from family, friends, and significant others can mitigate stress and BO, ultimately strengthening pandemic resilience and public health outcomes.

## 1. Introduction

According to the World Health Organization, COVID‐19 continues to be a significant public health issue [1]. The disease rapidly spread worldwide, resulting in COVID‐19 pandemic, with more than 778 million confirmed cases and nearly 7.1 million deaths [2]. Since the pandemic’s inception, Saudi Arabia has reported approximately 841,469 confirmed cases, with a total of 9646 deaths [2]. As confirmed by the Centers for Disease Control and Prevention [3], COVID‐19’s manifestations range from severe infection symptoms to mild infection without symptoms, causing extreme fear, anxiety, stress, depression, and burden among healthcare providers, particularly nurses, who work on the frontlines and face direct exposure to confirmed or suspected COVID‐19 patients. These stressors negatively influence their professional, personal, and family lives, compounded by ambiguity about their future [4, 5].

### 1.1. Theoretical Framework and Conceptual Model

The conceptual foundation of this study is rooted in Stamm’s [6] ProQOL model, which conceptualizes the dual nature of the “helper’s” experience when caring for individuals undergoing trauma or suffering. Within this framework, ProQOL is divided into two primary aspects: CS and compassion fatigue. CS represents the positive fulfillment and sense of efficacy derived from caregiving. Conversely, compassion fatigue serves as the negative counterpart, categorized into BO; characterized by exhaustion, frustration, and system‐related stress; and STS, which involves the vicarious emotional toll of being exposed to patient trauma.

**FIGURE 1:**
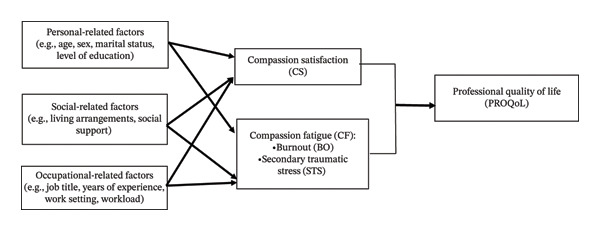
Conceptual framework of professional quality of life dimensions.

As illustrated in the conceptual framework (Figure 1), this study posits that the extreme conditions of the COVID‐19 pandemic acted as a significant environmental catalyst that influenced these three dimensions. The model identifies three categories of influence. First, predictor variables consist of demographic characteristics (age, gender, and living arrangements) and pandemic‐specific occupational stressors (high patient loads and personal COVID‐19 infection). Second, these predictors are hypothesized to directly impact the ProQOL constructs, with high‐demand factors such as infection risk and workload acting as primary drivers for the erosion of satisfaction and the escalation of fatigue.

Finally, the framework incorporates perceived social support (from family, friends, and significant others) as a protective mechanism based on the Social Buffer Hypothesis (see Figure 1). This hypothesis suggests that strong interpersonal networks serve as a “buffer” that can moderate the negative impact of workplace stressors, thereby protecting nurses’ mental well‐being and sustaining their ability to provide high‐quality care. This visual and theoretical representation aligns with the multivariate regression analysis used in this study to determine the independent weight of each factor while accounting for the complex, multifaceted nature of nursing work during a global health crisis.

According to ProQOL theory, exposure to infectious diseases, such as through contact with confirmed or suspected COVID‐19 patients, traumatized individuals, or disaster victims can significantly impact the ProQOL of nursing staff and other healthcare professionals [7]. This exposure may lead to positive outcomes such as fostering CS, which is the fulfillment and sense of purpose derived from helping others, feeling valued by colleagues, and contributing meaningfully to the workplace and society [6]. Conversely, negative consequences can occur such as CF, which encompasses two distinct components: BO and STS. BO reflects emotional exhaustion, hopelessness, and reduced efficacy in managing work demands, while STS arises from indirect exposure to trauma, manifesting as fear, and work‐related trauma [6].

Recent studies have emphasized that CF experienced by healthcare professionals is a form of traumatic stress, presenting as emotional and physical distress resulting from repeated exposure to traumatic events in the healthcare setting. Professionals under such stress may request more time off, face difficulty in continuing to do their jobs, or need to leave their profession due to feelings of helplessness and exhaustion, a lack of self‐satisfaction, a reduced ability to feel empathy, irritability, low concentration, pains, and aches, resulting in decreased professional effectiveness [8, 9]. Furthermore, BO is a significant contributing factor among nursing staff experiencing CF. It is characterized by detrimental shifts in attitudes and behaviors, including mental exhaustion, emotional detachment from work, reduced professional efficacy, and depersonalization. [6]. These symptoms often stem from an inability to cope with demanding workplace conditions and the persistent physical and psychological strain caused by the disconnection between nurses’ expectations of fulfilling their professional responsibilities and the realities imposed by organizational policies and structures, particularly during the COVID‐19 pandemic [10]. Research studies have confirmed that healthcare workers, particularly critical care nurses, experience the highest rates of BO syndrome, which is closely associated with the development of CF in high‐stress clinical settings [6, 11].

During pandemic outbreaks, STS often arises from healthcare professionals’ repeated exposure to patients who have endured severe psychological or physical trauma. The impact of STS can manifest in a range of debilitating symptoms, including fear of transmitting the virus to loved ones, feelings of helplessness, chronic fatigue, emotional exhaustion, sleep disturbances, and intrusive recollections of patients’ traumatic experiences. Numerous studies have identified STS as a predominant factor contributing to CF among healthcare workers across diverse healthcare settings, highlighting its critical role in shaping the emotional burden of frontline care [12].

Nurses have faced serious mental and physical challenges during the COVID‐19 pandemic. These challenges include stress, loneliness, fear of getting infected, and being separated from loved ones. Research shows that these problems can lead to anxiety and depression among healthcare workers. Over time, these effects can impact nurses’ ability to provide care. Nurses may also feel less satisfied with their work, which could cause them to leave their jobs or even leave the profession entirely [13].

Understanding the personal, social, and occupational factors influencing nurses’ well‐being is critical, especially during high‐stress periods such as the COVID‐19 pandemic. Empirical studies revealed a range of demographic, social, and occupational factors that contribute to healthcare professionals, especially the staff nurses’ exposure to extraordinary and traumatic situations within clinical settings. These include lack of skills, competencies, and knowledge deficits related to universal precautions, as well as inadequate training in infection control procedures. Additional stressors, such as improper use of personal protective equipment, understaffing, limited healthcare infrastructure, excessive workloads, extended working hours, low job satisfaction, restricted access to essential treatments, lack of social support, and feelings of inadequate support from their managers can result in traumatic events experienced in the clinical setting among frontline nurses and other healthcare workers [13, 14]. These predictors can significantly impact nurses’ professional functioning and the quality of care they provide.

Despite the growing body of international literature examining the ProQOL of nurses during the COVID‐19 pandemic, most studies have been conducted in Western or East Asian countries and have primarily focused on prevalence rates of BO, CF, and stress during peak periods of the pandemic, with limited attention to postwave experiences. Moreover, few studies have explored the combined influence of personal‐, social‐, and work‐related factors over time, resulting in gaps in understanding the sustained impact of the pandemic on nurses’ ProQOL. Research in Saudi Arabia remains scarce, particularly studies assessing nurses’ ProQOL after successive waves of COVID‐19. Considering Saudi Arabia’s unique healthcare system, multicultural nursing workforce and sociocultural context, examining ProQOL in the postwave period is essential to generate context‐specific evidence that can inform long‐term workforce support strategies and policy development. Identifying and predicting such determinants enables hospital administrators and stakeholders to design targeted support programs aimed at reducing STS and BO, while enhancing CS. These interventions not only promote physical and psychological well‐being but also improve job satisfaction and elevate the overall standard of patient care. Therefore, this study aimed to investigate the levels and predictors of ProQOL dimensions among nurses directly involved in the direct care of COVID‐19 patients.

## 2. Methods

### 2.1. Study Design

The study employed a cross‐sectional, descriptive, correlational design and followed the Strengthening the Reporting of Observational Studies in Epidemiology (STROBE) guidelines.

### 2.2. Participants and Samples

A purposive sample was drawn from registered nurses who had provided direct care to COVID‐19–positive patients in inpatient units and agreed to participate in this study. Exclusion criteria encompassed nursing students, nursing interns, and those with less than 1 year of clinical experience. Sample size estimation was performed using G∗Power software Version 3.1. Based on a significance level of 0.05, a statistical power of 0.95, an effect size of 0.15, and 11 predictors, the minimum required sample size for an inferential analysis was calculated to be 178. To compensate for potential attrition and missing data, the sample size was increased by 10%, yielding a final sample of 200 participants.

### 2.3. Data Collection

Data were collected from January to April 2024 using a self‐reported survey administered to nurses working in inpatient wards that received COVID‐19 patients at two governmental hospitals in Riyadh, Saudi Arabia. The researchers ensured that the participants met the inclusion criteria before data collection. The survey was created using Microsoft Forms, and targeted participants were invited to complete it by scanning a QR code and responding via their smartphones.

The survey consisted of three parts. The first part collected personal‐, social‐, and occupational‐related data from participants, including age, sex, educational level, job title, and living arrangement. The second part utilized the Multidimensional Scale of Perceived Social Support (MSPSS) [15], a 12‐item scale assessing individuals’ perception of their social support levels. This scale comprises three subdomains, namely, family, friends, and significant others, with four questions in each. Responses are rated on a 7‐point Likert‐type scale, ranging from 1 = “*very strongly disagree*” to 7 = “*very strongly agree*.” Scores are classified as low (12–35), moderate (36–60), and high (61–84), with higher scores indicating a greater level of perceived social support. The third part employed the ProQOL Scale (PROQOL‐5) [6], which includes 30 items divided into three subscales: CS, STS, and BO. Each subdomain contains 10 questions rated on a 5‐point Likert scale ranging from 1 = “*never*” to 5 = “*very often*.” Scores are classified as low (10–22), moderate (23–41), and high (42–50), with higher scores reflecting higher levels of CS, STS, and BO. Both tools have demonstrated validity and reliability in nursing populations. In this study, Cronbach’s α values for the MSPSS and its subdomains (family, friends, and significant others) were 0.932, 0.919, 0.913, and 0.916, respectively. For the PROQOL‐5 and its subscales (CS, STS, and BO), the Cronbach’s α values were 0.770, 0.911, 0.849, and 0.715, respectively, indicating acceptable to excellent internal consistency.

### 2.4. Data Analysis

The data were analyzed using IBM SPSS Statistics 28.0 for Windows. Descriptive statistics were utilized to compute frequencies and percentages for categorical variables and means with standard deviations for continuous variables, considering personal‐, social‐, and occupational‐related factors. The normality of differences in STS across these factors was assessed. Independent *t*‐tests and one‐way analyses of variance (ANOVAs) were performed to identify statistically significant differences in ProQOL dimension scores across personal‐, social‐, and occupational‐related factors.

Multiple linear regression models were employed to identify predictors influencing participants’ CS, STS, and BO. For each outcome, the initial full model included the following independent variables: personal characteristics (age, sex, education level, and marital status); social factors (living arrangements and perceived social support subdomains—family, friends, and significant others); and occupational variables: job title, years of experience, work setting, shift type, workload, and COVID‐19–related factors (number of patients, contact hours, and personal infection history). To refine the models, a backward elimination method was applied. The criterion for removal was set at a significance level of (*p* > 0.10) to ensure the retention of potentially relevant covariates while identifying the most robust predictors. Multicollinearity was monitored using variance inflation factors (VIFs), and a final *p* value of < 0.05 was required for a predictor to be considered statistically significant in the final model.

### 2.5. Ethical Considerations

Ethical approval for the study was secured from the Institutional Review Board (IRB) at (removed for peer‐reviewing). A digital consent form was presented on the survey’s first page, outlining the study’s purpose, what participation would entail, and participants’ right to withdraw at any time without consequences. No risks related to participation were identified. Consent was indicated by checking a box. Strict measures were taken to protect privacy and confidentiality, with no personal identifiers or sensitive information collected or retained.

## 3. Results

Table 1 illustrates the prevalence of ProQOL dimensions and perceived social support among the study participants. The mean ± SD was 90.13 ± 9.00 for the ProQOL, 32.69 ± 6.3 for CS, 29.17 ± 5.3 for STS, and 28.25 ± 4.3 for BO. Additionally, 63.5% of participants exhibited a moderate ProQOL, 67.5% experienced moderate CS, 58.5% had moderate levels of STS, and 53.5% reported a moderate level of BO.

**TABLE 1:** The scores of the professional quality of life and its dimensions among the study sample.

Measure	Professional quality of life (ProQOL)	Compassion satisfaction (CS)	Secondary traumatic stress (STS)	Burnout (BO)
	(*M* = 90.13, SD ± 9.00)	(*M* = 32.69, SD ± 6.3)	(*M* = 29.17, SD ± 5.3)	(*M* = 28.25, SD ± 4.3)
	*N* (%)	*N* (%)	*N* (%)	*N* (%)
Low	71 (35.5)	42 (21.0)	77 (38.5)	93 (46.5)
Moderate	127 (63.5)	135 (67.5)	117 (58.5)	107 (53.5)
High	2 (1.0)	23 (11.5)	6 (3.0)	0 (0.0)

Table 2 presents the personal demographic characteristics of the participants and their association with ProQOL dimensions. The age ranged from 22 to 64 years or older. The majority were female (81.5%), and half of the participants were single. Most participants (89.0%) held a bachelor’s degree. The univariate analysis of personal characteristics revealed that age significantly influenced both CS (*F* = 3.459, *p* = 0.046) and BO (*F* = 3.647, *p* = 0.028). Specifically, nurses in the 45–64 age group reported higher mean scores for CS (*M* = 34.09) and lower levels of BO (*M* = 26.23) compared to their younger age group. Gender‐based differences were also prominent, with female nurses reporting significantly higher levels of STS (*t* = 8.503, *p* = 0.004) and BO (*t* = 4.413, *p* = 0.037) than male nurses. Additionally, education level was significantly associated with STS (*F* = 6.921, *p* = 0.009), where nurses holding a diploma degree exhibited higher stress levels (*M* = 31.20) than those with bachelor’s or master’s degrees.

**TABLE 2:** Univariate analysis of the personal‐related factors associated with professional quality of life dimensions (*n* = 200).

Variables	*n* (%)	Compassion satisfaction (CS)		Secondary traumatic stress (STS)		Burnout (BO)	
		*M* ± SD	*t*/*F* (*p*)	*M* ± SD	*t*/*F* (*p*)	*M* ± SD	*t*/*F* (*p*)
Age (years)							
22–34	110 (55.0)	31.52 ± 5.90	*F* = 3.459^∗^	29.55 ± 5.29	*F* = 3.048	29.08 ± 3.96	*F* = 3.647^∗^
35–44	69 (34.5)	33.15 ± 6.63	(*p* = 0.046)	29.39 ± 5.19	(*p* = 0.050)	28.13 ± 4.56	(*p* = 0.028)
45–64	21 (10.5)	34.09 ± 5.64		26.47 ± 5.79		26.23 ± 3.87	
Gender							
Male	37 (18.5)	35.37 ± 7.72	*t* = 2.010	27.70 ± 5.66	*t* = 8.503^∗∗^	26.91 ± 5.81	*t* = 4.413^∗^
Female	163 (81.5)	32.07 ± 5.82	(*p* = 0.137)	29.50 ± 5.25	(*p* = 0.004)	28.57 ± 3.90	(*p* = 0.037)
Marital status							
Single	100 (50.0)	32.00 ± 7.07	*F* = 0.112	28.96 ± 4.95	*F* = 1.030	27.17 ± 4.77	*F* = 0.969
Married	76 (38.0)	33.13 ± 8.22	(*p* = 0.953)	27.72 ± 6.31	(*p* = 0.380)	27.00 ± 5.00	(*p* = 0.408)
Divorced/widowed	24 (12.0)	32.88 ± 6.59		32.50 ± 6.36		30.50 ± 2.12	
Level of education							
Diploma degree	12 (6.0)	31.91 ± 4.98	*F* = 0.103	31.20 ± 4.68	*F* = 6.921^∗∗^	29.00 ± 3.62	*F* = 1.971
Bachelor’s degree	178 (89.0)	32.72 ± 6.46	(*p* = 0.902)	29.12 ± 5.27	(*p* = 0.009)	28.33 ± 4.73	(*p* = 0.162)
Master’s degree	10 (5.0)	33.00 ± 5.57		28.16 ± 7.06		28.21 ± 4.38	

^∗^ *p* < 0.05.

^∗∗^ *p* < 0.01.

^∗∗∗^ *p* < 0.001.

Table 3 presents the social characteristics of the participants and their association with ProQOL dimensions. Among the participants, 41.5% resided with their families and 33.5% reported having vulnerable family members during the COVID‐19 pandemic. Furthermore, 51.5% expressed a fear of transmitting COVID‐19 to their family members, and approximately one‐third disclosed that their family members had contracted COVID‐19 due to the participant’s occupational exposure. In terms of perceived social support, on average, 41.5% of participants reported a moderate level of perceived social support, with 36.5% receiving moderate support from family and 45% from significant others, whereas 94% experienced low support from friends.

**TABLE 3:** Univariate analysis of the social‐related factors associated with professional quality of life dimensions.

Variables	*n* (%)	Compassion satisfaction (CS)		Secondary traumatic stress (STS)		Burnout (BO)	
		*M* ± SD	*t*/*F* (*p*)	*M* ± SD	*t*/*F* (*p*)	*M* ± SD	*t*/*F* (*p*)
Living arrangement status							
Alone	86 (43.0)	31.04 ± 5.27	*F* = 5.356^∗∗^	29.26 ± 3.75	*F* = 2.405	28.70 ± 2.91	*F* = 1.770
With family	83 (41.5)	34.03 ± 6.76	(*p* = 0.005)	27.32 ± 6.62	(*p* = 0.093)	27.00 ± 5.45	(*p* = 0.173)
With significant other	31 (15.5)	33.64 ± 6.93		29.77 ± 6.12		28.27 ± 5.05	
Having vulnerable family members during COVID‐19							
Yes	67 (33.5)	32.14 ± 5.46	*t* = 2.998	29.35 ± 4.98	*t* = 3.595	28.67 ± 3.84	*t* = 2.179
No	133 (66.5)	33.77 ± 7.69	(*p* = 0.085)	28.82 ± 6.08	(*p* = 0.050)	27.44 ± 5.16	(*p* = 0.141)
Afraid to transmit COVID‐19 to family							
Yes	103 (51.5)	33.46 ± 6.32	*t* = 3.230	29.88 ± 5.85	*t* = 3.753	28.39 ± 4.21	*t* = 1.971
No	97 (48.5)	31.86 ± 6.25	(*p* = 0.074)	28.42 ± 4.70	(*p* = 0.054)	28.14 ± 4.49	(*p* = 0.162)
Family infected with COVID‐19 because of your work							
Yes	66 (33.0)	31.61 ± 6.20	*t* = 0.050	29.53 ± 6.23	*t* = 6.329^∗∗∗^	28.38 ± 4.44	*t* = 5.381^∗∗∗^
No	134 (67.0)	32.83 ± 6.62	(*p* = 0.823)	28.00 ± 4.89	(*p* < 0.001)	28.01 ± 4.18	(*p* < 0.001)
Family support							
High	54 (27.0)	36.55 ± 7.01	*F* = 15.863^∗∗∗^	28.00 ± 6.41	*F* = 4.795^∗^	26.03 ± 5.75	*F* = 10.716^∗∗∗^
Moderate	73 (36.5)	31.26 ± 4.25	(*p* < 0.001)	29.57 ± 4.02	(*p* = 0.010)	28.94 ± 3.24	(*p* < 0.000)
Low	73 (36.5)	31.26 ± 6.39		29.64 ± 5.62		29.23 ± 3.51	
Friend support							
High	2 (1.0)	48.50 ± 0.707	*F* = 10.785^∗∗∗^	49.00 ± 1.41	*F* = 20.369^∗∗∗^	30.00 ± 0.00	*F* = 4.315^∗^
Moderate	10 (5.0)	37.70 ± 5.65	(*p* < 0.001)	33.20 ± 5.57	(*p* < 0.001)	29.00 ± 4.05	(*p* = 0.012)
Low	188 (94.0)	32.25 ± 6.07		28.75 ± 4.88		28.20 ± 4.39	
Significant other support							
High	48 (24.0)	37.29 ± 7.44	*F* = 19.911^∗∗∗^	27.45 ± 6.25	*F* = 4.052^∗^	25.18 ± 5.95	*F* = 18.806^∗∗∗^
Moderate	90 (45.0)	31.08 ± 4.06	(*p* < 0.001)	29.09 ± 4.66	(*p* = 0.019)	28.98 ± 3.07	(*p* < 0.001)
Low	62 (31.0)	31.34 ± 5.81		30.14 ± 5.12		29.41 ± 3.22	

^∗^ *p* < 0.05.

^∗∗^ *p* < 0.01.

^∗∗∗^ *p* < 0.001.

Significant variations in CS (*F* = 5.356, *p* = 0.005) were observed across living arrangements, with participants living with their families exhibiting higher CS (*M* = 34.03) compared to those living alone (CS: *M* = 31.04) or with roommates (CS: *M* = 33.64). Moreover, participants with vulnerable family members reported higher STS (*M* = 29.35) compared to those without vulnerable family members (*M* = 28.82), but the difference was not significant (*t* = 3.595, *p* = 0.050). Similarly, those fearing COVID‐19 transmission to their families demonstrated elevated STS (*M* = 29.88) compared to participants without such fears (*M* = 28.42), but the difference was not significant (*t* = 3.753, *p* = 0.054). Finally, participants whose family members were infected due to occupational exposure exhibited significantly higher STS (*t* = 6.329, *p* < 0.001, *M* = 29.53) and BO (*t* = 5.381, *p* < 0.001, *M* = 28.38) compared to participants whose family members were not infected due to their work (STS: *M* = 29.00, BO: *M* = 28.01). The results indicated statistically significant differences (*p* < 0.05 or *p* < 0.01) in mean ProQOL scores based on varying levels of perceived social support. Study participants reporting high family support levels displayed significantly higher CS (*M* = 36.55) and lower STS (*M* = 28.00) and BO (*M* = 26.03) compared to those with moderate or low levels of family support. Similarly, higher levels of support from friends were associated with significantly greater CS (*M* = 48.50) and reduced STS (*M* = 49.00) and BO (*M* = 30.00). Likewise, participants who reported high levels of support from significant others showed significantly elevated CS (*M* = 37.29) and lower STS (*M* = 27.45) and BO (*M* = 25.18).

Table 4 presents an overview of the distribution of the occupational‐related characteristics of the study participants and their association with ProQOL dimensions. The majority of participants were staff nurses categorized as Staff Nurse 1 (84.5%) and had over 5 years of professional experience (73%). Approximately half of the participants (53%) worked in noncritical care wards, while 47% were employed in critical care wards. A substantial proportion (84%) reported working 12‐h shifts. Regarding COVID‐19 patient care, 66% managed 1‐2 patients per shift, whereas 34% cared for three or more. Furthermore, 60% of the participants had less than 5 h of contact with COVID‐19 patients per shift, while 40% experienced contact exceeding 5 h. The majority (65%) reported contact with COVID‐19 patients across multiple shifts, and 46% of participants reported contracting COVID‐19 during the pandemic.

**TABLE 4:** Univariate analysis of the occupational‐related factors associated with professional quality of life dimensions.

Variables	*n* (%)	Compassion satisfaction (CS)		Secondary traumatic stress (STS)		Burnout (BO)	
		*M* ± SD	*t*/*F* (*p*)	*M* ± SD	*t*/*F* (*p*)	*M* ± SD	*t*/*F* (*p*)
Job title							
Staff Nurse 1	169 (84.5)	33.29 ± 6.47	*t* = 0.329	27.87 ± 6.49	*t* = 2.179	27.25 ± 5.37	*t* = 1.971
Staff Nurse 2	31 (15.5)	32.57 ± 6.31	(*p* = 0.567)	29.41 ± 5.11	(*p* = 0.141)	28.44 ± 4.13	(*p* = 0.162)
Years of experience							
Less than 5 years	54 (27.0)	31.88 ± 6.02	*t* = 0.665	29.70 ± 5.78	*t* = 9.141^∗∗^	28.41 ± 4.13	*t* = 4.717^∗^
More than 5 years	146 (73.0)	34.87 ± 6.65	(*p* = 0.416)	28.97 ± 5.21	(*p* = 0.003)	27.85 ± 4.91	(*p* = 0.011)
Work setting							
Noncritical wards	106 (53.0)	33.95 ± 6.46	*t* = 0.022	28.69 ± 5.10	*t* = 5.525	28.30 ± 3.69	*t* = 3.647^∗^
Critical wards	94 (47.0)	31.83 ± 6.11	(*p* = 0.883)	29.87 ± 5.68	(*p* = 0.020)^∗^	29.20 ± 5.19	(*p* = 0.028)
Workload (hours/shift)							
≤ 12 h	32 (16.0)	34.18 ± 7.92	*t* = 2.146	28.78 ± 7.11	*t* = 0.204	27.28 ± 5.78	*t* = 1.954
> 12 h	168 (84.0)	32.40 ± 5.96	(*p* = 0.145)	29.25 ± 4.98	(*p* = 0.652)	28.45 ± 4.02	(*p* = 0.164)
Number of COVID‐19 patients you cared for per shift							
1‐2	132 (66.0)	32.99 ± 6.36	*t* = 0.886	28.11 ± 5.39	*t* = 4.061^∗^	28.64 ± 4.03	*t* = 2.969
3 or more	68 (34.0)	32.10 ± 6.26	(*p* = 0.348)	29.71 ± 5.28	(*p* = 0.045)	27.52 ± 4.85	(*p* = 0.086)
Contact hours with COVID‐19 patients per shift							
< 5 h	120 (60.0)	33.51 ± 6.77	*t* = 1.609	27.16 ± 5.89	*t* = 3.753	27.81 ± 5.05	*t* = 3.216
> 5 h	80 (40.0)	31.45 ± 5.40	(*p* = 0.206)	29.18 ± 4.49	(*p* = 0.054)	28.93 ± 2.92	(*p* = 0.074)
Common shift during which contact with COVID‐19 patients occurred							
Morning	43 (21.5)	33.06 ± 7.05	*F* = 0.639	28.53 ± 6.13	*F* = 5.228^∗^	28.02 ± 4.59	*F* = 2.405
Night	27 (13.5)	32.34 ± 5.71	(*p* = 0.529)	29.51 ± 5.08	(*p* = 0.023)	27.74 ± 6.43	(*p* = 0.093)
Mixed	130 (65.0)	33.74 ± 7.89		28.55 ± 5.44		28.45 ± 3.72	
Infected with COVID‐19 during pandemic period							
Yes	92 (46.0)	30.31 ± 4.40	*t* = 27.147^∗∗∗^	29.69 ± 3.86	*t* = 5.356^∗∗^	29.63 ± 2.40	*t* = 18.202^∗∗∗^
No	108 (54.0)	34.71 ± 7.00	(*p* < 0.001)	28.73 ± 6.35	(*p* = 0.005)	27.10 ± 5.23	(*p* < 0.001)

^∗^ *p* < 0.05.

^∗∗^ *p* < 0.01.

^∗∗∗^ *p* < 0.001.

The univariate analysis showed that nurses with over 5 years of experience had significantly lower STS (*M* = 28.97, *t* = 9.141, *p* = 0.003) and BO (*M* = 27.85, *t* = 4.717, *p* = 0.011) compared to those with less than 5 years of experience (STS: *M* = 29.70, BO: *M* = 28.41). Additionally, nurses working in critical care settings reported higher STS (*M* = 29.87, *t* = 5.525, *p* = 0.020) and BO (*M* = 28.20, *t* = 3.647, *p* = 0.028) compared to those in noncritical settings (STS: *M* = 28.69, BO: *M* = 28.30). Nurses caring for three or more COVID‐19 patients per shift reported elevated STS (*M* = 29.71, *t* = 4.061, *p* = 0.045) compared to those caring for 1‐2 patients (*M* = 28.11). Similarly, prolonged contact (> 5 h per shift) with COVID‐19 patients was associated with higher STS (*M* = 29.18) compared to briefer contact (< 5 h per shift, *M* = 27.16), but the difference was not significant (*t* = 3.753, *p* = 0.054). Moreover, working night shifts was independently associated with increased STS (*M* = 29.51, *F* = 5.228, *p* = 0.023) compared to morning (*M* = 28.53) and mixed shifts (*M* = 28.55). Furthermore, participants who contracted COVID‐19 exhibited significantly lower CS (*M* = 30.31) and significantly higher STS (*M* = 29.69) and BO (*M* = 29.63) than those who did not contract COVID‐19 (CS: *M* = 34.71, STS: *M* = 28.73, BO: *M* = 27.10). These findings are supported by significant *t*‐statistics (CS: *t* = 27.147, *p* < 0.001; STS: *t* = 5.356, *p* = 0.005; BO: *t* = 18.202, *p* < 0.001).

The multivariate regression analysis reveals specific pathways through which demographic, occupational, and social factors influence ProQOL (Table 5). The final regression model showed that CS was lower among nurses who tested positive for COVID‐19, managed higher patient loads, and had family members infected due to their work. Conversely, CS was higher among those living with family and receiving healthy social support. Regarding STS, it was found to be higher among older nurses and those who had tested positive for COVID‐19, with the fear of transmitting the virus to family members acting as a primary stressor, while age was confirmed as a significant independent predictor specifically for traumatic stress, alongside the fear of transmitting the virus to family. For the BO dimension, testing positive for COVID‐19 remained the most prominent occupational risk factor. Across all models, perceived social support from all sources acted as a consistent and powerful buffer, both enhancing professional satisfaction and protecting against psychological erosion of BO and trauma.

**TABLE 5:** Regression analysis examining covariates of compassion satisfaction, secondary traumatic stress, and burnout.

Model	Compassion satisfaction (CS)^a^					Secondary traumatic stress (STS)^b^					Burnout (BO)^c^				
	*B*	SE	β	*t*	Sig.	*B*	SE	β	*t*	Sig.	*B*	SE	β	*t*	Sig.
(Constant)	49.268	6.692		7.362	0.000	10.927	6.089		1.795	0.074	15.049	4.966		3.030	0.003
Age	0.09	0.08	0.082	1.16	0.245	0.17	0.08	0.169	2.03	0.043	−0.04	0.05	−0.054	−0.82	0.412
Gender	2.602	1.114	0.160	2.335	0.021	2.145		0.156	2.124	0.035	—	—	—	—	—
Living arrangement during COVID‐19	1.229	0.639	0.139	1.924	0.049	—	—	—	—	—	—	—	—	—	—
Family afraid that you might transmit COVID‐19 to them	—	—	—	—	—	3.005	—	0.281	2.626	0.009	—	—	—	—	—
Family infected with COVID‐19 because of your work	−2.461	1.265	−0.183	−1.946	0.044	—	—	—	—	—	—	—	—	—	—
Number of COVID‐19 patients cared for per shift	−2.488	0.936	−0.187	−2.657	0.009	—	—	—	—	—	—	—	—	—	—
Positive COVID‐19 test	−4.716	0.922	−0.372	−5.114	0.000	0.816		−0.070	0.973	0.033	2.395		−0.275	3.499	0.001
Family support	2.195	0.088	0.186	2.244	0.032	−2.731	0.074	−0.168	−2.435	0.011	−2.712	0.070	−0.190	−2.387	0.010
Friend support	1.446	0.170	0.488	8.525	0.000	−1.454	0.142	−0.578	−10.229	0.000	−1.482	0.135	−0.582	−10.22	0.000
Significant other support	0.257	0.087	0.229	2.965	0.003	−0.284	0.073	−0.298	−3.904	0.000	−0.272	0.069	−0.351	−3.924	0.000

^a^ *F* = 3.871, *p* = 0.000, *R* ^2^ = 0.26, adjusted *R* ^2^ = 0.197.

^b^ *F* = 1.958, *p* = 0.016, *R* ^2^ = 0.155, adjusted *R* ^2^= 0.076.

^c^ *F* = 1.827, *p* = 0.028, *R* ^2^ = 0.146, adjusted *R* ^2^ = 0.066.

For analysis, categorical variables were dummy‐coded: age (Ref: 22–34 years), gender (Ref: male), living arrangement (Ref: living with family), and testing positive for COVID‐19 (Ref: No). Although the CS, STS, and BO models were statistically significant, the adjusted *R* ^2^ values (0.066–0.197) show that the predictors explain only 6.6%–19.7% of the variance in ProQOL dimensions. This indicates that a substantial portion of the variance remains unexplained, likely due to other unmeasured psychological or organizational factors.

## 4. Discussion

This study assessed the ProQOL among nurses caring for COVID‐19 patients and explored the personal‐, social‐, and work‐related factors that may influence it. The findings indicated that overall, nurses had moderate ProQOL, with approximately one‐third experiencing a low ProQOL. CS, STS, and BO were all reported at moderate levels, with CS being higher than both STS and BO. This pattern may explain the overall moderate ProQOL, as CS is known to buffer the negative effects of STS and BO. While none of the nurses reported high levels of BO, a small number exhibited high levels of STS—an alarming finding considering the decreasing number of COVID‐19 patients and the subsiding of major global waves. According to Stamm [6], for nurses to function effectively, CS should be high, while BO and STS should remain at moderate to low levels. In general, the ProQOL observed in this study aligns with the findings from previous studies conducted in Saudi Arabia [16], China [17], and the United States [18]. However, variations in CS, STS, and BO levels across these studies suggest that these factors are influenced by a combination of personal, social, and occupational variables.

Our study indicates that older nurses tend to report higher levels of CS and lower levels of STS and BO. This finding aligns with previous research conducted in Portugal [19] and Canada [20], showing that older nurses experience greater CS and reduced BO and STS. Older nurses often develop stronger clinical competence, confidence, resilience, and copying mechanisms with years of practice. Regarding gender differences, in our study, female nurses reported higher levels of STS and BO compared to their male counterparts. This contrasts with the findings from a similar study in Saudi Arabia [16], where gender did not significantly influence ProQOL scores. The higher emotional engagement and empathy exhibited by women may increase their susceptibility to STS and BO due to the emotional labor of nursing [6]. However, it is important to note that the proportion of female participants in our study was four times higher than that of male participants, necessitating cautious interpretation of these results.

The findings on social factors highlight that living with family increases CS, which is consistent with the literature. When a nurse lives with family, regular acts of shared responsibility and care within the family might reinforce their sense of purpose, kindness, and empathy [21, 22]. Conversely, those living with their loved ones might fear transmitting diseases to family members, especially those who are vulnerable, which can contribute to increased STS and BO levels. Although the differences were not significant in our study, such nurses demonstrated higher STS and BO. A third of nurses infected their family with COVID‐19 and demonstrated significantly higher STS and BO. This might be a result of the psychological and moral toll of the guilt and self‐blame for causing harm to a loved one and the added stress of caregiving responsibilities for a sick family member [23]. Higher BO levels can negatively affect nurses’ ProQOL, as well as the quality of care and patient health [24]. Our results also indicate that nurses with higher levels of support exhibit higher levels of CS and lower levels of STS and BO, which is in line with the literature [25]. In addition to support, family could be a great source of resilience and contribute to better well‐being and a more balanced work‐life experience for nurses [22, 25].

Regarding work‐related factors, the levels of STS and BO were higher among staff nurses with fewer years of experience and those working in critical care wards. This finding contrasts with a study in China [26], which may be due to differences in cultural context and sample characteristics. Novice nurses often lack the clinical exposure necessary to build confidence in their skills, decision‐making abilities, and resilience or effective coping strategies [27]. Consequently, they may experience heightened stress even during routine tasks and become easily overwhelmed by patient suffering and death, challenges that experienced nurses are generally better equipped to handle [28]. Working in critical care further intensifies these challenges due to the associated emotional labor and physical demands. The high acuity of patients, constant exposure to trauma, and frequent encounters with death place substantial psychological burdens on staff [29]. Notably, STS levels were found to be higher among nurses who cared for three or more COVID‐19 patients and those who worked night shifts. This finding is similar to a study in Italy [30], which reported that increased contact hours with COVID‐19 patients, as well as having family members or friends develop COVID‐19, were associated with higher levels of STS and emotional exhaustion. Furthermore, our study revealed that nurses who were themselves infected with COVID‐19 exhibited lower levels of CS, along with higher levels of both STS and BO.

Regarding predictors, we found that CS was influenced negatively by having a family member infected with COVID‐19 due to work, handling more COVID‐19 patients per shift, and testing positive for COVID‐19. STS was positively associated with gender, fear of COVID‐19 transmission to family, and testing positive for COVID‐19. Lastly, perceived social support was a predictor of CS, STS, and BO. It impacted CS positively while influencing STS and BO negatively. This means that as perceived social support from family, friends, and significant others increases, CS increases, and STS and BO decrease. This highlights the importance of social support in improving the ProQOL by increasing CS and decreasing STS and BO. Targeted interventions addressing these factors can play a crucial role in enhancing nurses’ ProQOL, as continued exposure without adequate and appropriate support may exacerbate feelings of exhaustion, fear, and trauma.

Although the statistical significance of the identified predictors, it is important to notice the relatively low explanatory power of the regression models, with adjusted *R* ^2^ values ranging from 0.066 to 0.197. This indicates that while significant factors were identified, other unmeasured variables (e.g., personality traits such as resilience, specific workplace culture, leadership quality, and access to mental health resources) likely play a major role in determining ProQOL. Future research should adopt a more holistic approach by incorporating these psychological and systemic variables to provide a more comprehensive understanding of the drivers of professional well‐being in nursing.

### 4.1. Strengths and Limitations

This study offers a comprehensive exploration of the personal, social, and occupational factors influencing nurses’ ProQOL during the COVID‐19 pandemic, providing valuable insights into CS, BO, and STS across diverse clinical settings. However, several limitations constrain the generalizability of these conclusions. A primary limitation is the use of a purposive sample with a relatively small sample size, drawn from two governmental hospitals within a single city. This strategy restricts the applicability of the results to the broader nursing population across different regions, private healthcare sectors, or varied clinical environments. Additionally, the cross‐sectional design prevents the establishment of causal relationships among variables such as social support, STS, and BO, which may fluctuate over time. The use of self‐report surveys may have also introduced social desirability bias or response set bias.

Furthermore, the low adjusted *R* ^2^ values in our regression models suggest a limitation in the study’s scope. While significant predictors were identified, a large portion of the variance in ProQOL remains unexplained. Our analysis did not account for unmeasured variables such as individual personality traits (e.g., resilience), specific workplace cultures, or the quality of nursing leadership. Consequently, the findings provide a valuable but partial view of the factors affecting nurses during the pandemic. Despite these constraints, the study highlights that enhancing CS is crucial for promoting quality patient care and mitigating BO and STS among frontline staff.

Based on these findings, future research should build upon these findings by expanding the geographic and institutional scope to include diverse healthcare settings. Longitudinal designs are essential to capture the evolving psychological impact of pandemics over time. Additionally, evaluating the specific role of peer support and institutional interventions, such as access to mental health resources and leadership training, might inform targeted strategies to safeguard nurses’ well‐being and reduce institutional costs associated with occupational stress.

## 5. Implications for Practice

The study highlights the multidimensional influence of personal‐, social‐, and work‐related factors on ProQOL of the nursing staff and broader healthcare organizational outcomes, making it a matter of critical concern. Based on our findings, special consideration should be directed toward female nurses and younger professionals, particularly those assigned to care for COVID‐19 patients in critical care settings during extended shifts, and who have limited experience in managing pandemic outbreaks. Moreover, nurses who tested positive for COVID‐19 and whose families were affected by occupational exposure exhibited elevated levels of STS and BO, along with reduced CS. Additionally, perceived social support serves as a protective factor, strongly associated with higher CS and reduced STS and BO. Therefore, it is imperious to develop tailored social support interventions that address their specific psychosocial needs of the nursing staff who are vulnerable to STS and BO during hard circumstances [31]. To cultivate a resilient and supportive work culture, healthcare organizations must prioritize the implementation of structured social support systems, including peer mentoring, team‐based debriefings, and access to psychological counseling that assist them to know how they can handle such health crises.

## 6. Conclusion

This study identifies several significant, albeit modest, associations between personal COVID‐19 infection, social support, and nurses’ ProQOL. While moderate levels of ProQOL were prevalent, the findings highlight that testing positive for the virus and managing high patient loads are critical factors that diminish CS and increase BO. Furthermore, older age and the fear of virus transmission emerged as specific drivers of STS. Conversely, social support from family and significant others served as a powerful consistent buffer across all dimensions. These results emphasize the need for healthcare systems to implement targeted interventions that not only reinforce social resilience but also address organizational stressors such as workload and infection risk. Strengthening these interpersonal networks and systemic support is essential to safeguarding nurses’ well‐being and sustaining high‐quality patient care during and after public health emergencies.

## Funding

No funding was provided for this study.

## Conflicts of Interest

The authors declare no conflicts of interest.

## Data Availability

The data that support the findings of this study are available on request from the corresponding author. The data are not publicly available due to privacy or ethical restrictions.
